# Contactless Microwave-Based Estimation of Complex Permittivity of Masonry Materials: A Frequency-Domain Approach

**DOI:** 10.3390/s26092693

**Published:** 2026-04-26

**Authors:** Zenon Szczepaniak, Paweł Juszczyński, Waldemar Susek, Krzysztof Tabiś, Zbigniew Suchorab

**Affiliations:** 1 Faculty of Electronics, Military University of Technology, Gen. Sylwestra Kaliskiego 2, 00-908 Warsaw, Poland; zenon.szczepaniak@wat.edu.pl (Z.S.); waldemar.susek@wat.edu.pl (W.S.); 2 Faculty of Mathematics and Information Technology, Lublin University of Technology, Nadbystrzycka 38, 20-618 Lublin, Poland; p.juszczynski@pollub.pl; 3 Aquapol Polska CPV Research Laboratory Krzysztof Tabiś, Stefana Żeromskiego 12, 58-160 Świebodzice, Poland; 4 Faculty of Environmental Engineering and Energy, Lublin University of Technology, Nadbystrzycka 40B, 20-618 Lublin, Poland; z.suchorab@pollub.pl

**Keywords:** microwave method, material moisture, moisture assessment, time domain, frequency domain, apparent permittivity, electrical conductivity, ceramic brick

## Abstract

This article concerns the issue of contactless estimation of the complex electrical permittivity of masonry materials by means of a microwave technique in the frequency domain. The main aim of the study was to develop a method enabling the determination of the real part of relative permittivity and the electrical conductivity of ceramic building materials using microwave reflection measurements, as well as to assess the applicability of the proposed approach for moisture diagnostics in porous media. The research was performed using a reflection-mode measuring setup comprising a vector network analyser and a broadband horn antenna, while measurements were carried out in the frequency range from 1 to 6 GHz on samples of solid ceramic brick with six gravimetric moisture levels. A one-dimensional model of electromagnetic wave propagation in the material was developed, considering complex permittivity, impedance transformation, and a calibration procedure compensating for the influence of the antenna and free-space propagation. Based on the fitting of the magnitude and phase characteristics of the reflection coefficient, the electrical parameters of the tested samples were estimated. The results obtained showed an increase in both permittivity and conductivity with increasing moisture content and revealed very good agreement with the reference values determined using the time-domain method. It can be concluded that the frequency-domain microwave approach may be effectively applied for contactless and non-destructive diagnostics and estimation of the dielectric properties and moisture content in ceramic materials.

## 1. Introduction

### 1.1. The Impact of Moisture on Building Structures

In the analysis of the properties of engineering materials, one of the key parameters remains the water content within their structures [1]. This quantity determines not only the physical state of the material but also its functional suitability. Therefore, its determination is an important element of material diagnostics [2]. The degree of moisture affects both the mechanical properties and the behaviour of the material under variable environmental conditions. From the perspective of building practice, this means that uncontrolled changes in moisture content may lead to reduced durability, deterioration of the service quality of building partitions, and a decline in the functional performance of the entire structure [3].

However, the importance of moisture is not limited solely to material-related issues. Elevated indoor air humidity is also a factor that worsens hygiene and health conditions in buildings. A particularly unfavourable situation occurs in buildings with insufficient air exchange, where limited ventilation combined with excess moisture promotes the growth of microorganisms [4]. This relationship had already been recognised in the nineteenth century, when the presence of mold fungi in buildings and their potential impact on the human body began to be described [5].

The effects of moisture are also reflected in the thermal performance of building envelopes. The penetration of water into insulating materials leads to an increase in the thermal conductivity coefficient and, consequently, to a reduction in their thermal insulation performance [6,7]. Since the thermal conductivity coefficient λ is derived from Fourier’s law, which forms the basis for describing steady-state one-dimensional heat flow [8], any change in its value directly affects the assessment of the energy performance of building partitions, and an increase in moisture content may result not only in material degradation but also in a deterioration of the thermal characteristics of the building.

### 1.2. Types of Moisture

The identification of the causes of moisture accumulation requires prior discussion of the basic physicochemical phenomena responsible for moisture exchange between a material and its surroundings. In this context, absorption, sorption, and desorption are of particular importance, as they describe the mechanisms by which building materials accumulate and release water [9,10,11,12,13].

Excessive moisture in building materials is one of the key operational challenges. The following sources of moisture are distinguished [14]:

- Capillary moisture—resulting from capillary rise of ground moisture;
- Condensation moisture—formed because of water vapor diffusion and its condensation on the surface of a partition;
- Flood moisture—associated with water absorption under conditions of excessive water presence in the surroundings;
- Installation-related moisture—caused by failures or leakage in water installations;
- Human-generated moisture—associated with various activities carried out by occupants, such as washing and drying;
- Technological moisture introduced into the building—resulting from transport, storage, and wet construction processes during building erection.

Among the mechanisms listed above, capillary rise is particularly important from the perspective of material degradation. Water rising through the material also transports dissolved salts, most commonly sodium chlorides and sulphates, into the partition. Their migration usually ends in zones of intense evaporation, where local ion accumulation and the formation of salt efflorescence occur [15]. The consequences of this process are twofold. On the one hand, the visual appearance of the surface is impaired; on the other hand, changes in the internal structure of the material occur. The precipitation of sparingly soluble compounds and their subsequent crystallisation within pores and capillaries increase internal stresses, which may lead to cracking, layer delamination, and damage to building elements [16].

Salts present in building partitions may originate both from natural processes and from human activities. An example of natural impact is the action of marine aerosol. Moisture particles containing salts, transported by wind, are deposited on building surfaces and may subsequently penetrate the material through cracks, leakages, damaged facade layers, or improperly executed insulation systems. Atmospheric precipitation further enhances this process. As a consequence, salts accumulate within the material structure, among which sodium chloride is most frequently identified, but sulphates and nitrates are also present [17]. This is of considerable importance in measurement analysis because dissolved salts increase the electrical permittivity of the material and may lead to overestimation of results interpreted as the degree of moisture content. At the same time, the presence of salts enhances the hygroscopicity of the material, promotes long-term moisture retention, and increases the susceptibility of the partition to further degradation [18].

### 1.3. Methods for Determining Moisture Content in Materials

The determination of moisture content in materials can be divided into two groups of methods, namely, direct and indirect methods [19]. The first group includes methods for direct moisture determination, among which the gravimetric drying method may be distinguished. This is one of the best methods for assessing the water content in a material because it is based on the direct comparison of the mass of a wet sample and a dried sample, which makes it possible to determine the moisture content accurately. However, the disadvantage of this method is the necessity of taking a sample from the building structure [20].

The second group consists of indirect methods, in which moisture content is determined based on other quantities that depend on the moisture level. In general, these methods are less accurate than direct methods [21,22]. This group includes, among others, optical-thermographic methods, in which the moisture content is determined on the basis of the observations of the investigated material’s temperature distribution. Their limitation is that only surface moisture can be determined [23].

In this place, it is especially worth mentioning indirect electrical methods. Among them, the most popular are resistance and capacitance methods. They are inexpensive but strongly influenced by disturbing factures such as ionic conductivity or temperature [24]. Against the background of the methods mentioned above, the microwave method compares favourably, as it eliminates most of the limitations described.

### 1.4. Electrical Permittivity of Materials

The application of electromagnetic methods for moisture assessment is based on the pronounced differences in dielectric properties between the individual phases present in a material. Water is of particular importance in this regard, as its molecule exhibits a permanent dipole moment. This results from its molecular structure: owing to its higher electronegativity, the oxygen atom attracts the shared electron pairs more strongly than the hydrogen atoms, leading to an asymmetric charge distribution [25]. In the presence of an external electromagnetic field, a reorganisation of this electronic structure occurs, resulting in changes in the dielectric response of the molecule.

The electrical properties of building materials should be analysed with consideration of their heterogeneous structure. Each material has a specific bulk density; however, the actual volume of the medium includes not only the solid phase but also pores filled with gas or liquid. Since each of these phases is characterised by a different relative electrical permittivity, the final response of the material to an electromagnetic field depends on its structure, porosity, and moisture content [26]. For air, the relative permittivity is assumed to be 1, whereas for water it is close to 80 [26].

Such a substantial difference between water and the remaining constituents of a material means that even a slight increase in moisture content may significantly alter the electrical properties of the tested medium. Recent data indicate that, at room temperature, the relative electrical permittivity of water is approximately 78 [27]. By comparison, ice reaches a value of about 3.15 [28], while dry snow, due to its high air content, typically falls within the range of 1.5–2.4 [28]. Rocks such as granite and sandstone are characterised by values on the order of 2–6 [29], whereas cement, mortar, and mature concrete usually reach values of approximately 3 to 5.5 [30,31,32]. In the microwave band, dried sulphate and carbonate minerals, including gypsum and calcite, exhibit values in the range of 6–9 [33,34]. These data indicate that the presence of water is the dominant factor affecting the change in the electromagnetic response of materials.

The dielectric properties of a material are described using the concept of complex relative permittivity [35]:

(1) $$\varepsilon_{r}=\varepsilon_{r}^{\prime }-i\varepsilon_{r}^{\prime \prime },$$

where *ε_r_* is the complex relative permittivity; *ε_r_′* is the real part of the relative permittivity; *ε_r_″* represents dielectric losses resulting from the delay in polarization response and conduction phenomena; and *i* is the imaginary unit (*i*^2^ = −1).

The real part describes the ability of the material to store electric field energy, whereas the imaginary part refers to energy dissipation and loss processes. In diagnostic practice, both components are important because they provide information on both the material structure and the presence of moisture and ionic admixtures.

### 1.5. Microwave Method

One of the methods used to assess moisture content in building materials is the microwave method, which is based on an analysis of the interaction between an electromagnetic wave and the investigated medium. In the measurement system, an antenna or a set of antennas is connected to a vector network analyser (VNA), which generates and receives the electromagnetic signal.

A complex overview of microwave methods for material property measurements and permittivity estimation may be found in refs. [36,37]. Further investigation of advancements in the field of microwave measurement techniques for materials, especially construction materials, will guide to the following areas of the interest:

- Open-ended probe, where a section of open-ended transmission line (usually a coaxial line) is used as a kind of contact sensor [38] for measuring the reflection coefficient and further calculating the permittivity of a material in contact with the opened end of the line;
- Resonance-based method, where a resonant cavity or planar resonant circuit is used for sensing the permittivity of a material sample placed inside the cavity or in contact with the planar version of the resonance circuit. Here, the concept is to measure a shift in the resonance frequency and change in a quality factor and then derive the sample permittivity value [39,40,41,42,43];
- Transmission methods are based on the measurement of reflection and transmission coefficients, usually the complete scattering matrix, most often by means of a vector network analyser operating in a two-port configuration. The sample may be placed in a waveguide or measured in free space. An alternative approach involves a time-domain analysis of the pulses emitted, reflected, and transmitted through a sample. In free-space systems, the measurement setup additionally includes two antennas, and the method allows the investigation of materials of practically arbitrary dimensions without extracting samples and placing them in a waveguide [44,45,46,47,48,49,50,51,52];
- Reflection methods are based on the measurement of the reflection coefficient, typically using a vector network analyser in a one-port configuration, with the sample placed either in a waveguide or in free space. The analysis may be performed either in the time domain, using signals reflected from the front and back surfaces of the sample, or in the frequency domain, based on the reflection coefficient. In free-space systems, the setup requires only one antenna and enables measurements of materials of arbitrary size without the need to extract samples or place them in a dedicated waveguide arrangement [53,54]. An interesting variant of this approach is microwave ellipsometry, in which two antennas are used and the reflected wave incident at an oblique angle onto the material surface is analysed [55];
- Radar-based methods form a separate, although methodologically related, group. These methods employ radar systems, most commonly ground-penetrating radar (GPR), to scan the investigated material and determine its internal layered structure as well as to estimate electrical permittivity. Such techniques require movement of the radar relative to the surface of the tested object and use dedicated GPR signal-processing techniques or synthetic aperture radar (SAR) methods [56,57,58].

Previous studies confirm the usefulness of microwave methods for the assessment of moisture content and dielectric properties of building materials, including masonry elements and porous materials [59,60]. At the same time, the available literature is predominantly focused on transmission-based systems employing two antennas, whereas studies using a single antenna in a reflection configuration remain scarce.

It follows that the level of moisture can be assessed based on an analysis of signal attenuation and phase variation. The validity of this approach is directly related to the very high electrical permittivity of water, which, compared to most building materials, markedly modifies the propagation conditions of a microwave signal [35]. This means that even a relatively small increase in moisture content may cause a sufficiently large change in the electromagnetic response of the material, which can be exploited in diagnostic applications.

Various interpretation techniques can be used to interpret microwave signals. A commonly used and relatively easy-to-analyse method is the time-domain method, which allows for an analysis of the velocity of signal propagation, and based on this, an estimation of the permittivity of the tested wet material. The second approach is the analysis of broadband microwave signals in the frequency domain, which requires more complex interpretation algorithms [26]. However, because it allows for signal interpretation across a wide frequency range, it allows for a more comprehensive analysis, considering the effects of both moisture and salinity. This article presents a method for assessing the electrical parameters of ceramic materials with varying degrees of moisture in the frequency domain.

## 2. Materials and Methods

### 2.1. Concept of Measurement Test Stand

**Figure 1:**
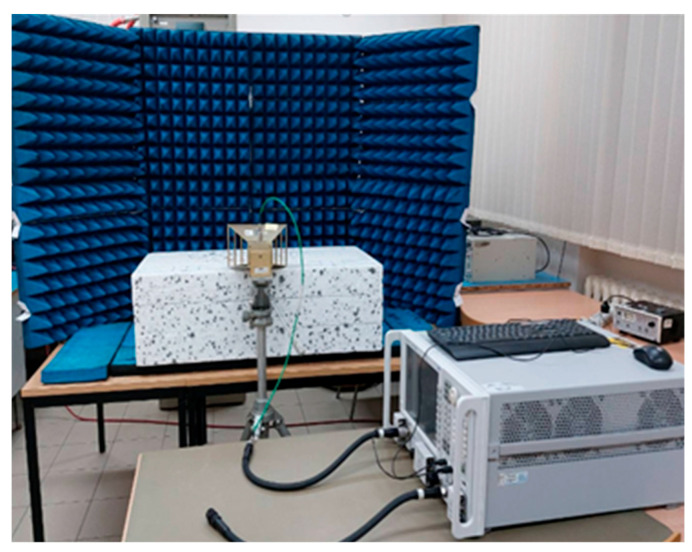
Photograph of the reflection-mode measurement setup used in the study.

The measurement setup used for investigating material samples in the reflection-mode configuration is shown in Figure 1.

The measurement setup consisted of: (1) a vector network analyser, Agilent N5224A (Agilent Technologies, Santa Clara, CA, USA); (2) a double-ridged horn antenna, A. H. Systems SAS-571 (A. H. Systems Inc., Chatsworth, CA, USA); and (3) microwave absorbers visible in Figure 1. The absorbers placed behind the tested object were used to suppress echo signals other than those originating from the object under investigation. The measurements were carried out over the frequency range of 1 to 6 GHz.

The broadband antenna was connected to only one VNA port. The use of this reflection-mode configuration enabled contactless and non-destructive measurement of the material sample under test.

The primary instrument used in the measurements was a vector network analyser (VNA), which enabled the generation and reception of microwave signals over a wide frequency range [61]. The next step involved selecting a broadband directional antenna capable of transmitting and receiving signals within the frequency band of interest while providing adequate directional gain. The directional characteristics of the antenna made it possible to maximise the power radiated toward the investigated sample and to improve the reception of low-power echo signals returning from the tested object [62]. An additional advantage of directional antennas is their low sensitivity to signals other than those originating from the sample under investigation [63].

Another advantage of the reflection method is that access is required from only one side of the tested object. The use of a single transmitting–receiving antenna reduces the sensitivity of the system to the wave propagation path in the investigated medium, since it eliminates the problem of proper mutual positioning of two antennas. The disadvantage of such an arrangement is that the reflected signal returning to the measurement system must propagate through the tested object twice. In the case of highly lossy materials, this leads to a low signal level at the receiver of the measurement setup. A signal propagating through an object and encountering a change in permittivity caused by the transition from the tested material to the air is reflected and returns through the same object to the measurement system [64]. To compensate for this limitation, either a higher transmitted power or an antenna with higher directional gain may be applied.

The antenna was positioned 8 cm from the sample surface and oriented normally to the tested brick face. The center of the antenna aperture was aligned with the center of the sample to ensure repeatable measurement conditions. The relative positions of the antenna and the sample were kept constant during the measurements. The free-space and metal-plane calibration measurements were performed at the same antenna distance as that used during the measurement of the tested samples. In the metal-plane calibration, a conductive plate larger than the antenna aperture was placed in front of the antenna at a distance of 8 cm. The VNA settings used during the measurements, including the frequency sweep range, number of frequency points, IF bandwidth, output power, and averaging conditions, were kept constant for all measurements.

### 2.2. Samples

Microwave tests were performed on 30 solid ceramic bricks with dimensions of 25.0 × 12.0 × 6.0 cm and an apparent density of 1800 kg/m^3^. The bricks were dried to an air-dry state in a laboratory dryer and then gradually saturated with water to obtain various moisture levels determined gravimetrically according to the PN-EN ISO 12570 standard: 5 samples of each of the following level moistures: 0% (air-dry state), 2% and 3% (low moisture level), 5% and 6% moisture (medium moisture level) and 9% moisture (high moisture level).

The gravimetric moisture content of the samples was determined according to Equation (2) [65]:

(2) $$w=\frac{m_{meas}-\;m_{dry}}{m_{dry}}\cdot 100\%,$$

where *w* is the gravimetric water content, *m_meas_* is the weight of the wet sample, and *m_dry_* is the dry mass of the sample.

### 2.3. Permittivity Estimation Approach

**Figure 2:**
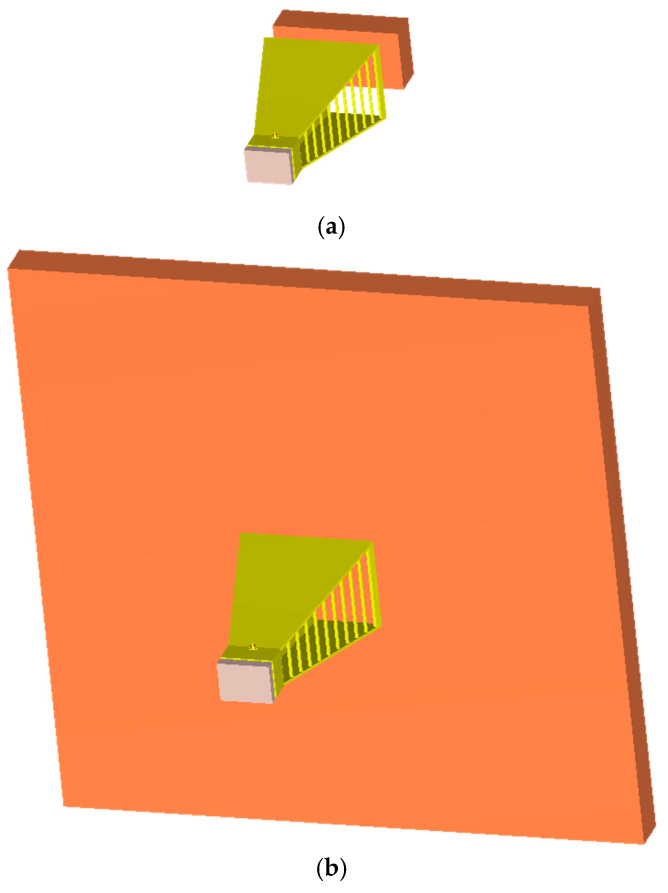
Simulation view for: (**a**) a single brick; (**b**) a wall.

Prior to developing the model, simulations of antenna performance, were carried out for two cases in CST Studio Suite (Dassault Systèmes, Vélizy-Villacoublay, France), namely, a single brick and a large wall with a specified electric permittivity. Figure 2 presents screenshots illustrating the course of the simulation.

**Figure 3:**
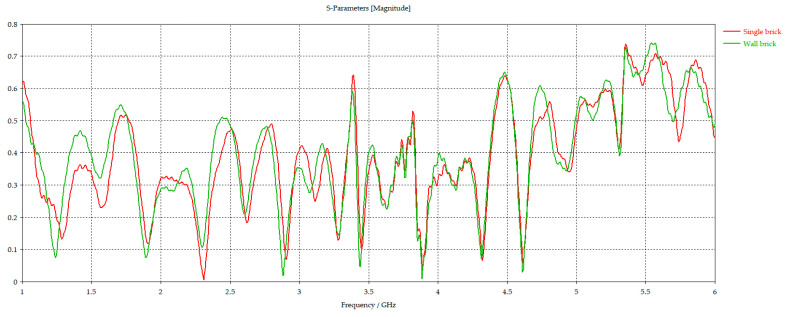
Comparison of the reflection coefficient for a single brick, marked in red, and a brick wall, marked in green.

The simulated magnitude data of the reflected signal S_11_, presented in Figure 3, showed only a slight influence of the investigated object size on the measurement results.

Therefore, the single-brick model was adopted for further analysis. The obtained absolute value of the mean difference in reflectance was 0.008, and the standard deviation was 0.062, indicating that the finite transverse dimensions of the brick had only a limited effect on the measured response in the adopted measurement configuration. It is worth mentioning that the single-brick model was selected because it made it easier to determine and maintain a uniformly distributed moisture content compared to a large wall and, consequently, to estimate the electric permittivity.

#### 2.3.1. Determination of Apparent Permittivity Using Time-Domain Method

To compare the ability of microwave-assisted permittivity estimation of wet ceramic materials in the frequency domain, analyses were performed in both the time and frequency domains. Time-domain permittivity readings are defined in this paper as the reference results and were determined based on signal reflections, which was presented in more detail by the authors of this paper in ref. [26,66]:

(3) $$\varepsilon ={\left(\frac{c\cdot \;t_{p}}{2L}\right)}^{2}$$

where *ε*—apparent permittivity of tested material, *t_p_*—time of MV signal travel along the measured ceramic sample [s], *c*—light velocity in vacuum [m/s], and *L*—thickness of the ceramic sample (0.06 m).

It should be noted that the time-domain method was used only to determine the apparent permittivity, i.e., the real part of the dielectric response of the material. Electrical conductivity was not determined using the time-domain method and was estimated exclusively using the frequency-domain microwave approach.

The determination of apparent permittivity using the frequency method is presented in detail in Section 2.3.2, Section 2.3.3 and Section 2.3.4.

#### 2.3.2. Methodology of Apparent Permittivity Determination Using the Frequency-Domain Method

After analysing the possible configurations of the measurement system, the reflection measurement formula was adopted. This means that an EM wave with a frequency in the optimal range determined based on the conducted research was sent toward the investigated object. Next, the returning wave was received, comprising reflections from the incident wave from the object surface (the air–object interface), structural discontinuities inside the object, and the object–air interface on the opposite side of the object. Since the measurement system received the returning wave, the natural microwave parameter used as the measurement result was the complex reflection coefficient. Laboratory measuring instruments, such as a vector network analyser (VNA), record this parameter using the scattering matrix convention, i.e., as S_11_ [67]. Therefore, to model the structure of the building partition and building material samples in a manner compatible with the measurements, calculations of the microwave reflection coefficient were applied.

The essence of modelling the properties of a material sample, or partition, involves calculating the complex reflection coefficient as a function of frequency and comparing its values with the values determined by a measuring device within the same frequency range. The analytically determined reflection coefficient contains dependencies and takes into account the influence of complex electric permittivity [68]. A model created in this way makes it possible to extract the value of the complex electric permittivity for the measured material sample by means of parameter optimisation and fitting the simulation results to the measurement results.

The principle of using the measured reflection coefficient involves comparing the values of its magnitude and argument with the corresponding values of the calculated reflection coefficient [69,70]. Subsequently, complex electric permittivity values are found that maximise agreement between the measured and simulated frequency characteristics.

For small material samples, for which the homogeneity of the material and the moisture distribution inside the sample may be assumed, the simulation and extraction procedures may concern the effective equivalent electric permittivity for the entire volume of the sample material. In that case, the single simulated layer is the material layer with a thickness corresponding to the physical thickness of the sample [71].

**Figure 4:**
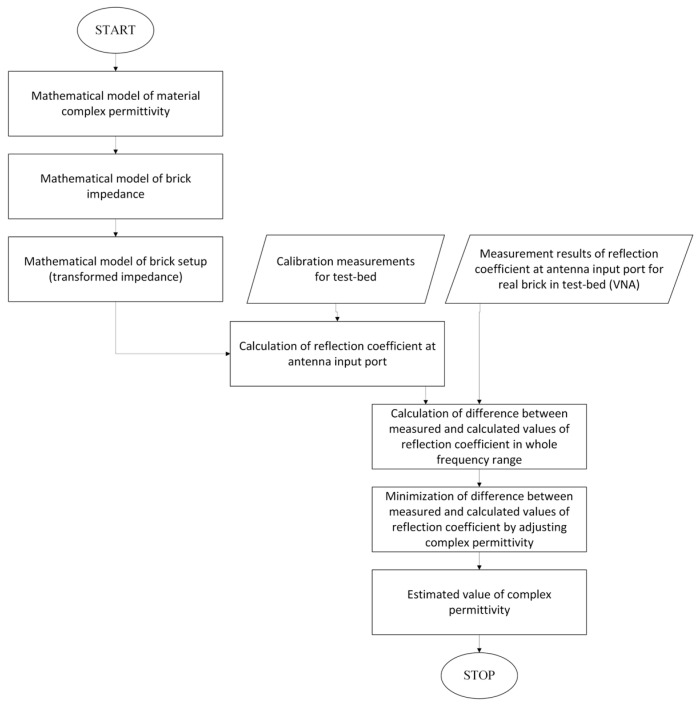
Methodology for estimating the permittivity from measured data using frequency domain approach. Oval shapes denote the start and end points of the procedure, rectangular boxes represent computational/modeling steps, and parallelograms indicate measurement or input data.

Figure 4 presents the procedure for determining the complex electric permittivity of the investigated object.

#### 2.3.3. Assumed Model of Material Permittivity

Investigations of electrical parameters were carried out on a building material, namely a brick, with different moisture levels.

The electrical properties of the medium from which the investigated object is made are described by the complex permittivity. At this stage of modelling, a relationship taking into account two parameters was adopted: *ε_r_*, that is, the real part of the relative permittivity, and σ, that is, the conductivity expressed in S/m (4) [72]. In the adopted approach, the imaginary part of complex permittivity was represented indirectly by the conductivity-dependent term, whereas dielectric relaxation effects were not considered explicitly. For the purpose of the analysis, it was assumed that the material from which the brick was made was a nonmagnetic dielectric medium, which means that the relative permeability was equal to 1 [73].


(4) $$\varepsilon_{r}={\varepsilon_{r}}^{\prime }-i\frac{\sigma }{2\pi f\varepsilon_{0}}$$

It should be noted that the adopted material model was a simplified approximation. In particular, the real part of permittivity was treated as an independent frequency within the analysed band, while the dielectric losses were represented only by an effective conductivity term. Such an approach does not explicitly account for dielectric dispersion and relaxation phenomena, which may become relevant for wet porous materials. Therefore, the estimated parameters should be interpreted as effective values valid within the considered frequency range. In the present study, this simplification was adopted deliberately in order to verify the usefulness of a limited-parameter model for practical frequency-domain estimation.

#### 2.3.4. Model of EM Wave Propagation in Measured Medium

The developed one-dimensional model of the propagation properties of the structure for an electromagnetic wave was based on the relationship describing the transformation of load impedance through a section of a lossy transmission line. The load impedance is the impedance of the system composed of the preceding layers, that is, the layers located farther from the sensor, whereas the lossy transmission line is represented by the currently analysed layer.

During modelling of the brick, the following parameters were taken into account: the intrinsic impedance of the brick material (4) and the wave propagation coefficient (6) [74]. The brick was treated as a one-dimensional medium having a length in the direction of wave propagation equal to the brick thickness *L*:

(5) $$Z=\sqrt{\frac{\mu }{\varepsilon }}=\sqrt{\frac{\mu_{0}}{\varepsilon_{0}\varepsilon_{r}}}$$

(6) $$\gamma =j\omega \sqrt{\mu \varepsilon }=j\omega \sqrt{\mu_{0}\varepsilon_{0}\varepsilon_{r}}$$

In this way, for the purpose of further wave propagation modelling, the brick material was treated as a virtual TEM waveguide.

**Figure 5:**
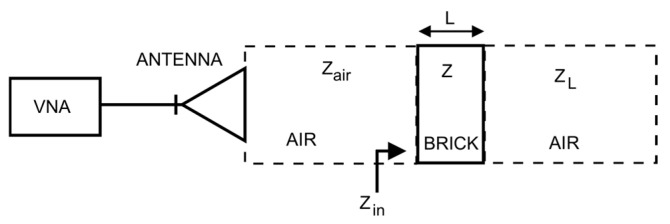
Transmission-line approach for modelling layered media: brick material and free-space behind it. Air layers in three-layer (air-brick-air) medium are marked in a dashed line.

For further considerations, it was assumed that the brick with a thickness of *L* = 0.06 m was a section of transmission line in which the wave propagated, whereas the air behind it, treated as an infinite layer, was the line termination with impedance *Z_L_* equal to the free-space impedance, that is, *Z_air_* = 120π Ω. This situation is illustrated in Figure 5.

The input impedance of a transmission line section terminated with impedance *Z_L_* can be calculated using the standard formula given below, which takes into account the effects of impedance transformation phenomenon (7) [75]:

(7) $$Z_{in}\left(L\right)=Z_{0}\frac{Z_{L}+Z_{0}\cdot tgh(\gamma L)}{Z_{0}+Z_{L}\cdot tgh(\gamma L)}$$

In Equation (5), *Z*_0_ is the characteristic impedance of the transmission line and *Z_L_* is the loading impedance placed at the end of the line. By substituting into Equation (7) the relationships obtained from Equations (5) and (6), the following expression is obtained (8):

(8) $$Z_{in}\left(L\right)=Z\frac{Z_{air}+Z\cdot tgh(\gamma L)}{Z+Z_{air}\cdot tgh(\gamma L)}$$

Next, the value of the reflection coefficient at the brick surface to the air interface can be expressed by means of impedance (8) in the form of Equation (9):

(9) $$\Gamma_{brick}=\frac{Z_{in}-Z_{air}}{Z_{in}+Z_{air}}$$

#### 2.3.5. Calibration of Antenna Parameters and EM Wave Propagation Effects

The measurement system comprising a VNA, which was previously calibrated, and an antenna was used to measure the value of the reflection coefficient at the antenna port. The values of the reflection coefficient should be interpreted as describing a complex measurement system composed of a brick placed in free space and located at a specified distance from the antenna [76]. It is not possible to directly interpret the measured value of the reflection coefficient at the antenna port as the reflection coefficient expressed by Equation (9). In addition, there is an obvious influence of the distance between the brick and the antenna on the value of the measured reflection coefficient.

Therefore, it was necessary to perform a compensation procedure for the influence of the antenna parameters and the wave propagation phenomena along the antenna to object path.

**Figure 6:**
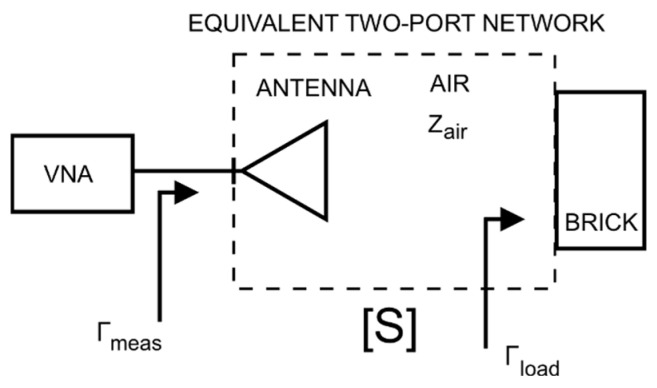
Concept of modelling the antenna and free-space propagation as an equivalent two-port network, marked with a dashed line.

This model considered the impedance of the brick placed in free space, but it did not consider the antenna reflection coefficient or the signal transmission from the port to the antenna aperture radiating the wave. To solve this problem, the influences of the antenna and wave propagation along the antenna to the brick path were modelled in the form of a two-port network described by the scattering matrix S. This equivalent two-port network concept is presented in Figure 6.

The reflection coefficient at port no. 1 of the two-port network described by the scattering matrix S, with a one-port network described by the reflection coefficient $\Gamma_{load}$ connected to port no. 2, is given by the following relationship, which can be determined using the signal flow graph technique:

(10) $$\Gamma_{meas}=S_{11}+\frac{S_{12}\cdot S_{21}}{1-S_{22}\cdot \Gamma_{load}}\cdot \Gamma_{load}$$

Here, *S*_11_, *S*_12_, *S*_21_, and *S*_22_ are adequate scattering parameters of the scattering matrix [*S*].

This virtual two-port network accounted for the influences of the transmission properties, impedance matching of the antenna, and wave propagation over the distance between the antenna and the surface of the investigated object (round trip). For further analysis, it was assumed that this two-port network was reciprocal, that is, *S*_12_ = *S*_21_. In addition, since, in general, port no. 2 of this two-port network corresponded to the plane of the air-to-air medium interface in the absence of the tested material, the assumption *S*_22_ = 0 was adopted.

**Figure 7:**
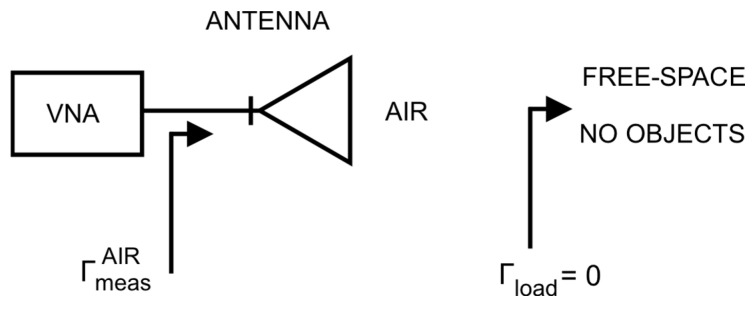
Scheme of the free-space calibration measurement.

**Figure 8:**
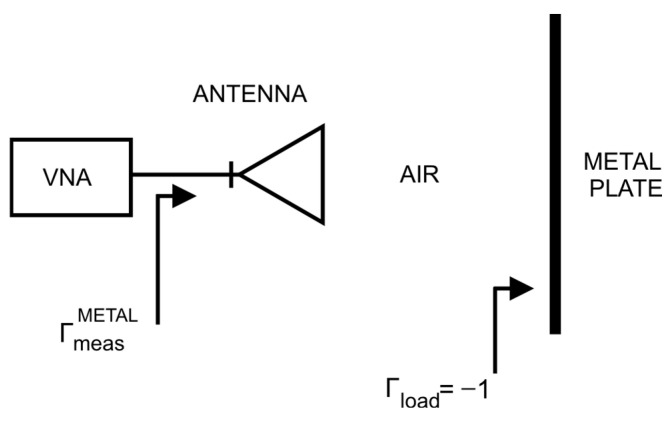
Scheme of the conducting plane calibration measurement.

Therefore, to fully describe the two-port network, only two scattering parameters remained to be determined, namely, *S*_11_ and *S*_21_. For this purpose, two calibration measures were carried out:

- Free space (Figure 7);
- Conducting plane (Figure 8).

As a result, the following expressions were obtained:

(11) $$\Gamma_{meas}^{AIR}=S_{11}+\frac{S_{21}^{2}\cdot 0}{1-0\cdot 0}=S_{11}$$

(12) $$\Gamma_{meas}^{METAL}=S_{11}+\frac{S_{21}^{2}\cdot \left(-1\right)}{1-0\cdot \left(-1\right)}=S_{11}{-S}_{21}^{2}$$

This made it possible to transform Equation (10) into the following form:

(13) $$\Gamma_{in}^{calc}=\Gamma_{meas}^{AIR}+\left(\Gamma_{meas}^{AIR}-\Gamma_{meas}^{METAL}\right)\cdot \Gamma_{brick}$$

The application of Equation (13) made it possible to calculate the reflection coefficient at the input port of the antenna when the reflection coefficient of the brick, given by Equation (9), and the two calibration parameters determined from the calibration measurements were known. The value of the reflection coefficient measured by means of the VNA, previously calibrated using a standard method, for example Short Open Load, in the real measurement system comprising the VNA, antenna, and brick, had the same reference plane, that is, the input port of the antenna. This meant that it was possible to compare the magnitudes and angles of the reflection coefficient obtained via brick measurements and the values of the reflection coefficient determined via calculations by modelling the impedance of the brick material according to Equation (13). Further, by adjusting the complex permittivity value in the mathematical model, the difference between the measured and calculated values was minimised and, finally, the value of the estimated complex permittivity was found.

## 3. Results

### 3.1. Reference Data

The results of the permittivity readings obtained using the time-domain method from 30 tested bricks characterised by 6 different moisture levels are presented in Table 1 and, in accordance with the nomenclature presented earlier, they are marked as reference readings.

The real part of the permittivity was determined within the same measurement experiment. The VNA used in the study enabled measurements in the time domain, which allowed the acquisition of time-domain waveforms. Based on the recorded signals, the time delay between peaks corresponding to reflections from sample boundaries was determined. Subsequently, the real part of the permittivity was calculated according to Equation (2), where L denotes the thickness of the tested sample.

### 3.2. Estimated Real Part of Relative Permittivity Using Frequency Domain Analysis

Subsequently, the frequency characteristics of the modulus and argument of the measured and calculated reflection coefficients were determined using Equations (4)–(9) and (13).

Minimisation of the difference between the measured and calculated results was performed using a deterministic grid-search procedure. The measured and modelled values of the calibrated complex reflection coefficient S_11_ were compared over the entire analysed frequency range. Since S_11_ is a complex quantity, the fitting error at each frequency point was defined as the modulus of the difference between the measured and calculated complex values, thus simultaneously accounting for both magnitude and phase. The total fitting error was obtained by summing these pointwise differences over all frequency samples.

Parameter estimation was carried out sequentially. First, the real part of the relative permittivity was fixed at ε′ = 3, and the conductivity σ was scanned in the range of 0 to 2 S/m with a step of 0.01 S/m. The value of σ corresponding to the minimum total fitting error was selected. Next, this optimal conductivity value was kept fixed, and ε′ was scanned in the range of 3 to 20 with a step of 0.1. The value of ε′ yielding the minimum total fitting error was taken as the final estimate.

This fitting approach was a simple exhaustive parameter scan rather than a gradient-based, evolutionary, or other stochastic optimisation method. Therefore, within the assumed search ranges and discretisation steps, the obtained solution was deterministic, repeatable, and robust with respect to initialisation.

**Figure 9:**
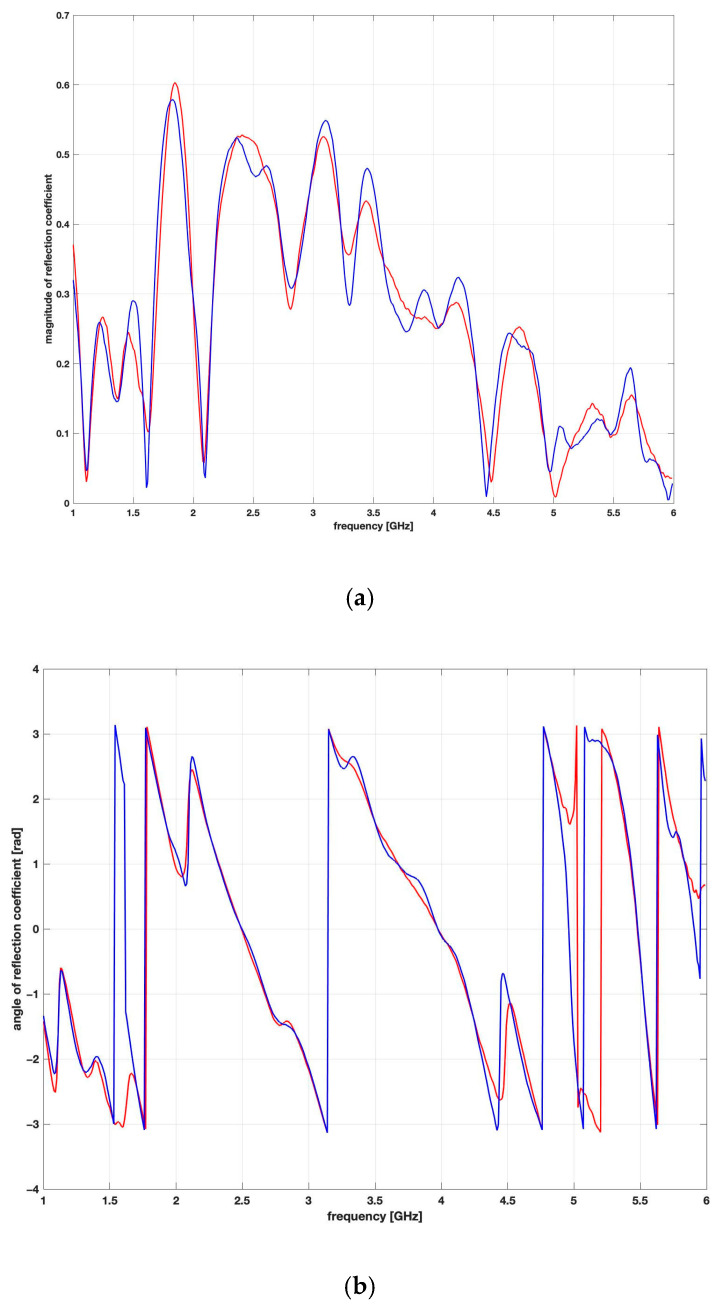
Exemplary comparison of reflection coefficients obtained via measurements (red) and calculations (blue) for (**a**) magnitude; (**b**) for angle [rad].

Figure 9 presents a common plot of the reflection coefficient for an exemplary brick with a mass moisture equal to 3%. The figure compares the frequency characteristics of the modulus and argument of the measured and calculated reflection coefficients according to Equation (10) to visualise the situation of maximum fitting of these characteristics for the optimal value of the estimated permittivity.

Table 2 summarises the estimated values of permittivity (ε′) and Table 3 summarises the conductivity (*σ*) values for the tested set of bricks. Since the fitting procedure was performed using the measured reflection coefficient characteristics over the whole frequency range of 1 to 6 GHz, the reported values of ε′ and σ should be treated as effective band-equivalent parameters of the simplified model.

## 4. Discussion

**Figure 10:**
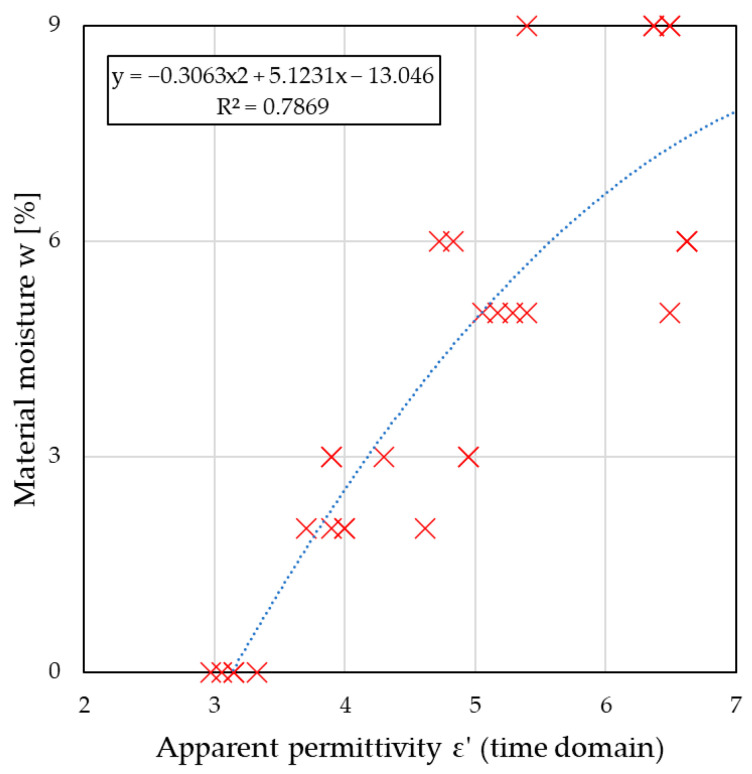
The relationship between the electric permittivity determined in the time domain and the moisture content of the material. Red × markers indicate the measured data points, and the blue dashed line represents the fitted trend line.

Analysis of the results obtained in the time domain showed an increase in permittivity with increasing sample moisture content, which confirmed that the measurements were performed correctly. Figure 10 shows the relationship between the permittivity readings and the moisture content determined gravimetrically, and the regression equation describing this relationship is defined.

The mathematical relationship presented in Figure 10 represents the relationship between the moisture content and the measured permittivity and can be used to calibrate a microwave setup for determining the moisture content of ceramic materials using the time-based method. The obtained coefficient of determination value of R^2^ = 0.787 indicated a very good fit between the model and the measurement data obtained. These data yielded RMSE = 1.3435 and MAE = 1.07045.

**Figure 11:**
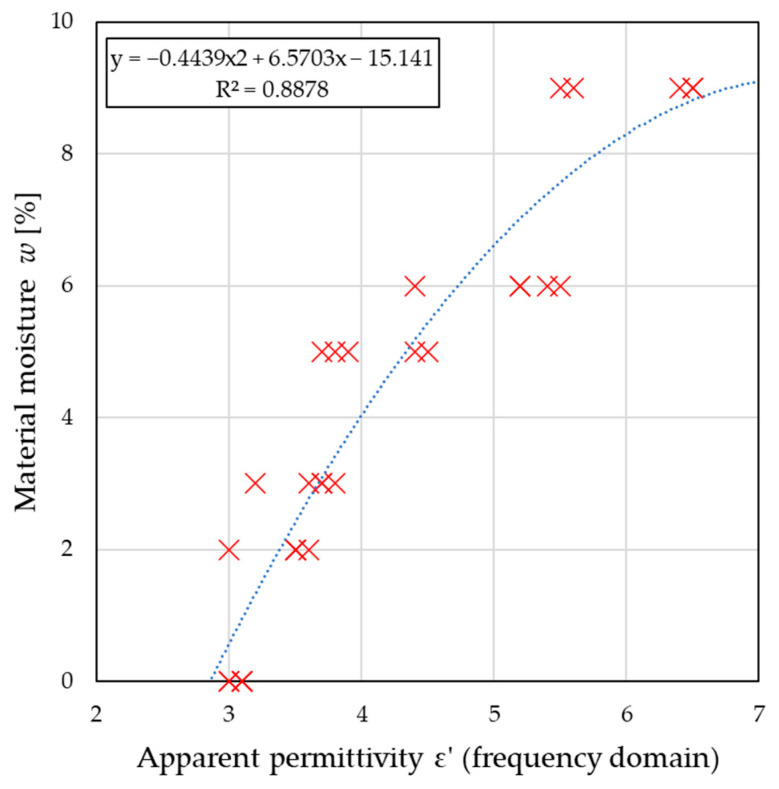
The relationship between the electric permittivity determined in the frequency domain and the moisture content of the material. Red × markers indicate the measured data points, and the blue dashed line represents the fitted trend line.

**Figure 12:**
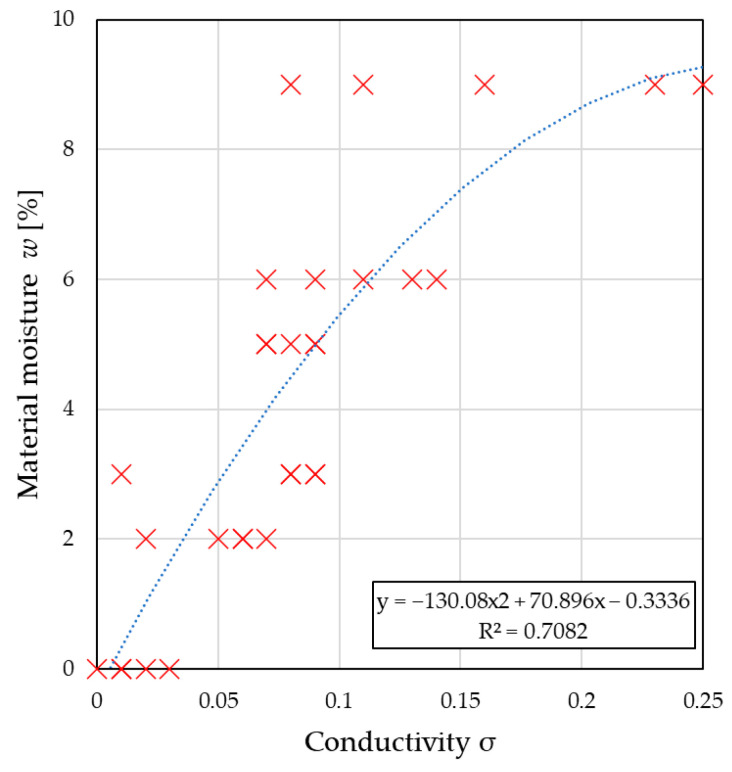
The relationship between the conductivity determined in the frequency domain and the moisture content of the material. Red × markers indicate the measured data points, and the blue dashed line represents the fitted trend line.

In turn, Figure 11 and Figure 12 present the relationships between the ε′ and σ coefficients obtained using the frequency-domain method.

As in the previous case, the mathematical models presented in the graphs provide calibration equations for estimating material moisture content based on permittivity and conductivity readings estimated in the frequency domain and can be used to calibrate microwave devices. The moisture prediction model developed using permittivity data achieved RMSE = 0.9751, MAE = 0.8175, and R^2^ = 0.888, which indicated a good fit and confirmed that the adopted model described the studied phenomenon well. In the case of conductivity, the model performance was lower, with R^2^ = 0.708, RMSE = 1.5723, and MAE = 1.2279.

**Figure 13:**
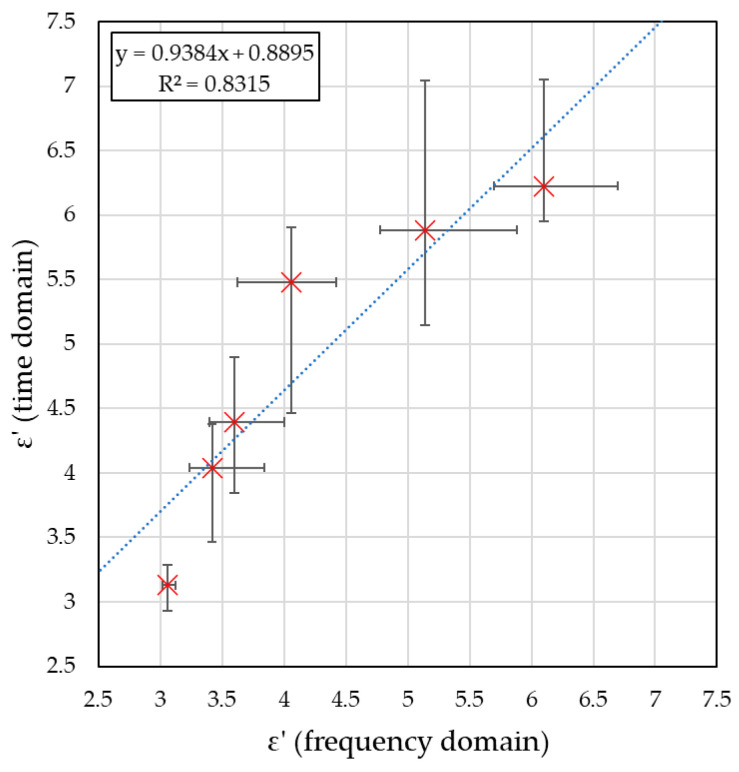
Graph showing the dependence between permittivity values measured using the use time-domain method and estimated using the frequency-domain method for the same set of materials. Red × markers indicate the measured data points, and the blue dashed line represents the fitted trend line.

Figure 13 shows the relationship between the estimated and reference permittivity values along with the fitted linear regression line. This relationship is described by a linear equation given by the Formula (14):

(14) y=0.9384x+0.8895

where *x* denotes the estimated permittivity value determined using the frequency-domain method, and *x* corresponds to the reference value obtained using time-domain analysis. As shown in the figure, the coefficient of determination R^2^ = 0.8315 indicates good agreement between the linear model and the experimental data. This means that the estimated permittivity values were in very good agreement with the reference values determined using the time-domain method. Moreover, the observed trend line shifted toward the permittivity values obtained using the time-domain method, which suggested that the microwave-based method may overestimate the electrical permittivity of ceramic brick.

**Figure 14:**
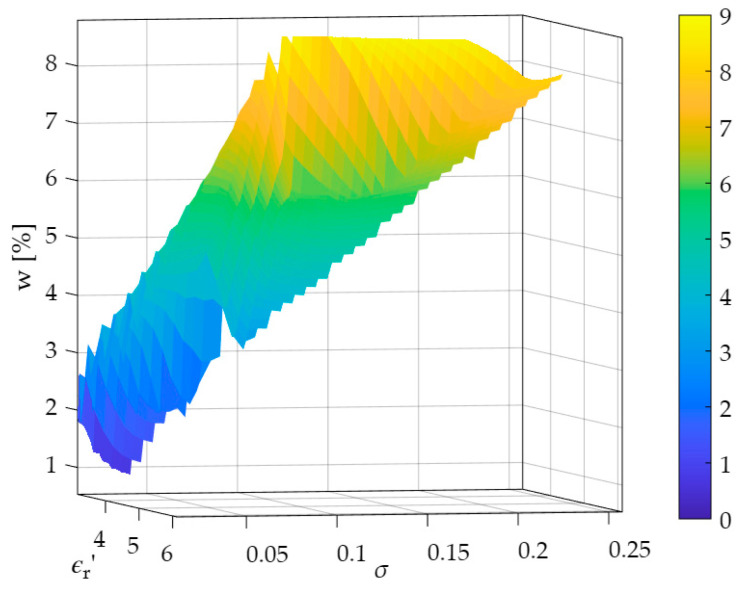
Graph showing the dependence of moisture content on ε‘ and σ estimated using the microwave frequency-domain method.

Figure 14 presents a three-dimensional graph illustrating the effect of a moisture content increase on the estimated value of electrical conductivity σ in relation to the estimated value of electrical permittivity ε′ using the frequency-domain method.

The presented analysis shows that with increasing material moisture content, both the value of electrical permittivity increases, which is related to the polar structure of the water molecule, and also the value of electrical conductivity, which is a consequence of the release of salt ions in water.

Based on the data obtained, a multiple regression model was developed that allows for microwave-based moisture determination in ceramic materials with varying salt ion contents. By accounting for the influence of different frequency ranges on the readings, this model accounts for the influence of salt ions present in moisture on the estimation, which is a significant advantage over time-based methods and other measurement techniques used to indirectly detect the presence of water. This equation takes the following form:

(15) $$w=2.631617\varepsilon^{\prime }-3.99431\sigma -6.638871$$

In this case, the moisture prediction model developed using combined permittivity and conductivity data achieved R^2^ = 0.86424, RMSE = 1.0725, and MAE = 0.9056. These results indicated that the combined model performed worse than the individual models, including the time-domain approach.

Comparing the obtained results of permittivity estimation, the results are similar or better than those reported in the literature. Achieving a coefficient of determination (R^2^) of 0.888 is satisfactory for the permittivity model determined using the microwave method. For example, Abdelsamei et al. compared two field methods for determining the permittivity of asphalt pavements, using the SR horn antenna and SR PaveScan, and compared the results to those obtained in the laboratory using the TOF method for cores. Similar coefficients of determination were obtained (R^2^ = 0.83 for the horn antenna and 0.82 for PaveScan), which were worse than the results obtained herein [77]. In the study by Juan et al., three microwave sensors were compared for indirectly determining changes in dielectric properties in the same blood plasma samples. The authors demonstrated that the sensors used in their study provided a high model fit to the measured data, obtaining an adjusted R^2^ > 0.85, which was comparable to the results obtained using the measurement method proposed in ref. [78]. To relate the quality of the obtained model to the team’s previous studies, the values of the coefficient of determination (R^2^) were compared. In the paper by Suchorab et al. [79], a value of R^2^ = 0.986 was obtained. In turn, in the study by Futa et al. from 2023, concerning the calibration of surface TDR sensors with the use of covariance analysis, R^2^ values ranging from 0.9820 to 0.9887 were obtained, with a mean value of 0.9850. This indicated a good fit of the adopted model to the measurement data, as well as an improvement in the quality of the description of the investigated relationship in comparison with the results presented in ref. [80].

The frequency-domain method proposed in this work is very promising and allows for an accurate estimation of moisture content in porous media using microwave methods. Further research is planned using a set of bricks with specific salinity levels in the analyses and developing models that will allow for estimation of not only the moisture content but also the salt ion concentration in the studied materials. For such a task, more advanced models of permittivity, such as the Debye formula, are planned. Then, the frequency dispersion and relaxation frequency of saltwater solutions will be modelled.

## 5. Summary and Conclusions

There are three possible methods for non-contact microwave measurements: resonance, transmission, and reflection. Each of them has advantages and disadvantages depending on the specific measurement conditions and application. The first one, the resonance method, requires highly accurate laboratory equipment and is used for testing small samples or samples with a specific shape. For the type of measurements considered in this work, its use would be impractical.

The proposed frequency-domain reflection-type microwave approach proved effective for the contactless estimation of dielectric parameters of ceramic masonry materials in the moisture range of 0% to 9%. Both estimated parameters showed a clear monotonic increase with increasing moisture content: the real part of relative permittivity rose from 3.00 for air-dry samples to 6.40 for samples with 9% moisture, while conductivity increased from 0.01 S/m to 0.25 S/m. This behaviour is consistent with the known dependence of dielectric properties on moisture content and serves here as a validation of the proposed reflection-mode frequency-domain approach. Moreover, the frequency-domain estimates were in very good agreement with the reference time-domain results, as confirmed by the regression equation y = 0.9384*x* + 0.8895 and the high coefficient of determination (R^2^ = 0.832), which validated the accuracy of the proposed method.

In this study, measurements were performed using the opacity method and a microwave antenna, which allows for non-contact measurement. After obtaining the measurement results from the VNA for the test sample, it was necessary to perform calculations to compensate for the antenna’s influence and the effects of wave propagation between the antenna and the sample. The compensation method presented in this article is simple and effective, minimising the influence of the antenna’s reflectivity and the distance between the antenna and the test sample. This requires preliminary calibration measurements in a very simplified configuration. Only two reflectivity measurements are required: one for the antenna directed into free space (measurement without objects) and one for a metal surface (plate) positioned at the target sample distance. The developed methodology for measuring and estimating permittivity allows for further research, including more complex modelling of complex permittivity by incorporating dispersion models such as Debye and Cole–Cole. Various advanced permittivity models for saltwater solutions may be implemented to develop algorithms for estimating their percentage content.

## Figures and Tables

**Table 1:** Apparent permittivity values determined using the reference method (time domain) of individual samples with different moisture levels.

Moisture [%]	Apparent Permittivity					
	Sample 1	Sample 2	Sample 3	Sample 4	Sample 5	Mean
0	3.06	3.15	3.15	3.33	2.97	3.12
2	3.99	3.99	3.89	3.70	4.61	4.04
3	3.89	3.89	4.29	4.94	4.94	4.39
5	5.28	5.40	5.06	5.17	6.49	5.48
6	4.83	4.72	6.62	6.62	6.62	5.88
9	6.49	6.37	6.49	6.37	5.40	6.22

**Table 2:** Estimated real part of complex relative permittivity using the frequency-domain method for a set of test materials.

Moisture [%]	Estimated ε′					
	Sample 1	Sample 2	Sample 3	Sample 4	Sample 5	Mean
0	3.1	3.1	3.1	3.0	3.0	3.06
2	3.5	3.5	3.5	3.0	3.6	3.42
3	3.7	3.7	3.6	3.2	3.8	3.6
5	3.9	3.8	4.4	3.7	4.5	4.06
6	5.2	5.4	5.5	4.4	5.2	5.14
9	6.4	6.5	6.5	5.5	5.6	6.1

**Table 3:** Estimated real part of complex relative conductance using the frequency-domain method for a set of test materials.

Moisture [%]	Estimated σ					
	Sample 1	Sample 2	Sample 3	Sample 4	Sample 5	Mean
0	0.01	0.02	0.01	0.00	0.03	0.014
2	0.05	0.06	0.06	0.02	0.07	0.052
3	0.08	0.09	0.08	0.01	0.09	0.070
5	0.07	0.07	0.09	0.08	0.09	0.080
6	0.11	0.13	0.14	0.07	0.09	0.108
9	0.23	0.16	0.25	0.08	0.11	0.166

## Data Availability

The data presented in this study are available upon request from the corresponding author.
